# Supplementary material for: Causal Relationship between Aging and Anorexia Nervosa: A White-Matter-Microstructure-Mediated Mendelian Randomization Analysis
Source: Biomedicines. 2024 Aug 16;12(8):1874. doi: 10.3390/biomedicines12081874 (PMC11351342; doi:10.3390/biomedicines12081874)
Supplement: Supplementary file 1 [file biomedicines-12-01874-s001.zip › Supplementary Figure/Figure S4/Singe SNP plot of MR analysis for TL to ubm-b-1950.pdf]

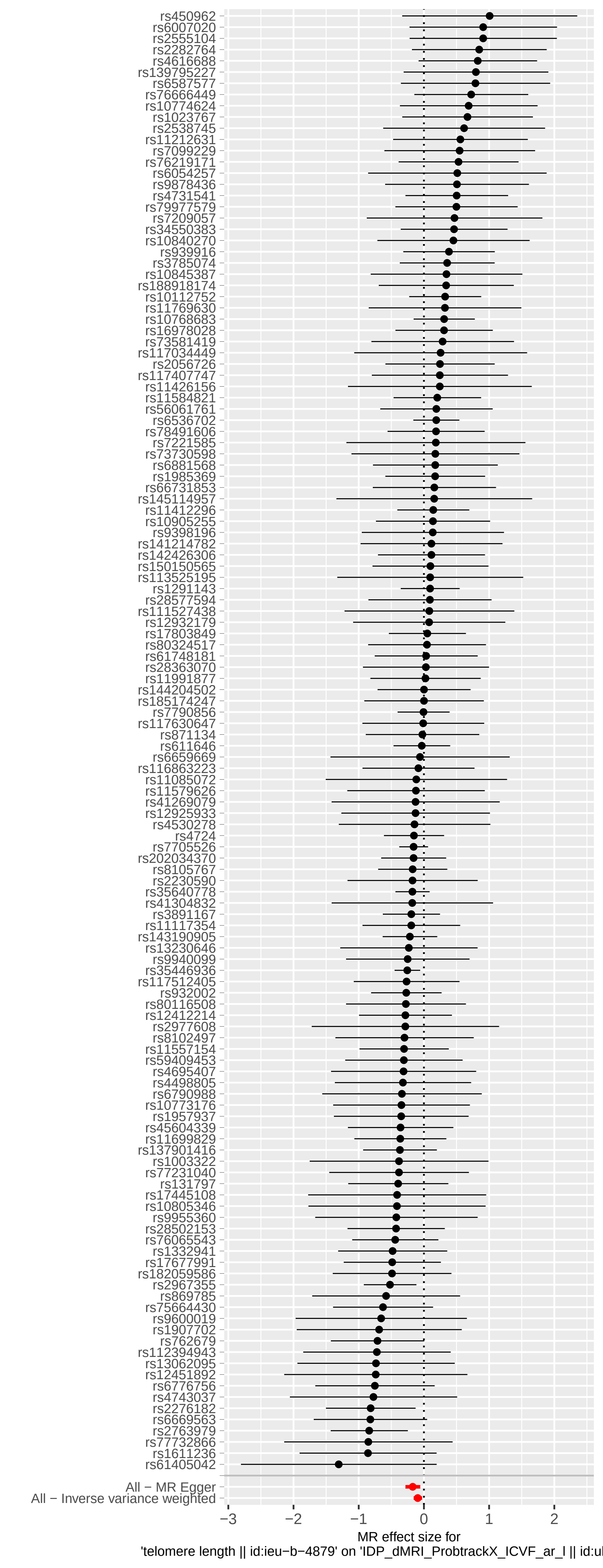

rs450962
rs6007020
rs2555104
rs2282764
rs4616688
rs139795227
rs6587577
rs76666449
rs10774624
rs1023767
rs2538745
rs11212631
rs7099229
rs76219171
rs6054257
rs9878436
rs4731541
rs79977579
rs7209057
rs34550383
rs10840270
rs939916
rs3785074
rs10845387
rs188918174
rs10112752
rs11769630
rs10768683
rs16978028
rs73581419
rs117034449
rs2056726
rs117407747
rs11426156
rs11584821
rs56061761
rs6536702
rs78491606
rs7221585
rs73730598
rs6881568
rs1985369
rs66731853
rs145114957
rs11412296
rs10905255
rs9398196
rs141214782
rs142426306
rs150150565
rs113525195
rs1291143
rs28577594
rs111527438
rs12932179
rs17803849
rs80324517
rs61748181
rs28363070
rs11991877
rs144204502
rs185174247
rs7790856
rs117630647
rs871134
rs611646
rs6659669
rs116863223
rs11085072
rs11579626
rs41269079
rs12925933
rs4530278
rs4724
rs7705526
rs202034370
rs8105767
rs2230590
rs35640778
rs41304832
rs3891167
rs11117354
rs143190905
rs13230646
rs9940099
rs35446936
rs117512405
rs932002
rs80116508
rs12412214
rs2977608
rs8102497
rs11557154
rs59409453
rs4695407
rs4498805
rs6790988
rs10773176
rs1957937
rs45604339
rs11699829
rs137901416
rs1003322
rs77231040
rs131797
rs17445108
rs10805346
rs9955360
rs28502153
rs76065543
rs1332941
rs17677991
rs182059586
rs2967355
rs869785
rs75664430
rs9600019
rs1907702
rs762679
rs112394943
rs13062095
rs12451892
rs6776756
rs4743037
rs2276182
rs6669563
rs2763979
rs77732866
rs1611236
rs61405042
All − MR Egger
All − Inverse variance weighted
−3
−2
−1
0
1
2
MR effect size for
'telomere length || id:ieu−b−4879' on 'IDP_dMRI_ProbtrackX_ICVF_ar_l || id:ul
